# Dichloridobis{6-methyl-2-[(trimethyl­silyl)amino]pyridine-κ*N* ^1^}cobalt(II)

**DOI:** 10.1107/S1600536809027937

**Published:** 2009-07-22

**Authors:** Xiaoyan Xue, Xia Chen, Hongbo Tong

**Affiliations:** a School of Chemistry and Chemical Engineering, Shanxi University, Taiyuan 030006, People’s Republic of China; b Institute of Applied Chemistry, Shanxi University, Taiyuan 030006, People’s Republic of China

## Abstract

In the structure of the title compound, [CoCl_2_(C_9_H_16_N_2_Si)_2_], the Co^II^ atom is located on an inversion center in a slightly distorted tetra­hedral environment formed by two chloride ions and the pyridine N atoms of two chelating 6-methyl-2-[(trimethyl­silyl)amino]pyridine ligands. The dihedral angle between the planes of the pyridine rings is 80.06 (5)°. Cohesion within the crystal structure is accomplished by N—H⋯Cl hydrogen bonds.

## Related literature

For the chemistry of *N*-functionalized amino ligands, see: Liddle & Clegg (2001 ▶); Engelhardt *et al.* (1988 ▶); Kempe (2000 ▶) and references therein. Trimethyl­silyl-substituted methyl pyridine ligands have been developed due to their structural features and good catalytic activity, see: Andrews *et al.* (2004 ▶).
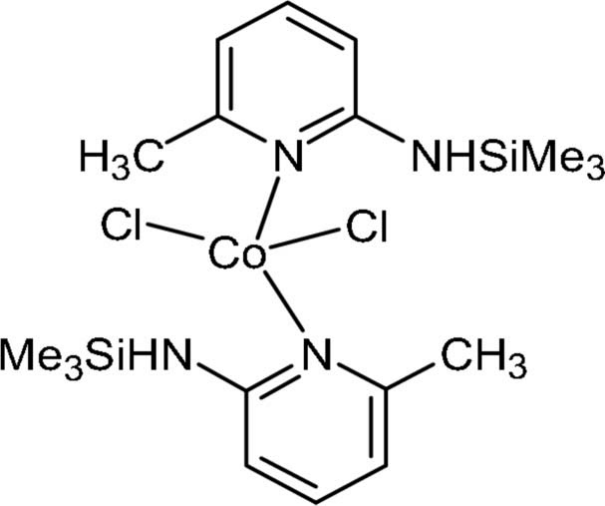


## Experimental

### 

#### Crystal data

[CoCl_2_(C_9_H_16_N_2_Si)_2_]
*M* *_r_* = 490.49
Monoclinic, 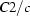

*a* = 14.817 (3) Å
*b* = 12.554 (4) Å
*c* = 14.886 (2) Å
β = 114.09 (2)°
*V* = 2527.8 (10) Å^3^
*Z* = 4
Mo *K*α radiation
μ = 1.00 mm^−1^
*T* = 213 K
0.30 × 0.30 × 0.20 mm

#### Data collection

Bruker SMART APEX CCD area-detector diffractometer
Absorption correction: multi-scan (*SADABS*; Sheldrick, 2004 ▶) *T*_min_ = 0.754, *T*_max_ = 0.826
5113 measured reflections
2224 independent reflections
1905 reflections with *I* > 2σ(*I*)
*R*_int_ = 0.020

#### Refinement

*R*[*F* ^2^ > 2σ(*F* ^2^)] = 0.033
*wR*(*F* ^2^) = 0.089
*S* = 1.02
2224 reflections
127 parameters
H-atom parameters constrained
Δρ_max_ = 0.47 e Å^−3^
Δρ_min_ = −0.20 e Å^−3^

### 

Data collection: *SMART* (Bruker, 1996 ▶); cell refinement: *SAINT* (Bruker, 1996 ▶); data reduction: *SAINT*; program(s) used to solve structure: *SHELXS97* (Sheldrick, 2008 ▶); program(s) used to refine structure: *SHELXL97* (Sheldrick, 2008 ▶); molecular graphics: *SHELXL97*; software used to prepare material for publication: *SHELXTL* (Sheldrick, 2008 ▶).

## Supplementary Material

Crystal structure: contains datablocks I, global. DOI: 10.1107/S1600536809027937/fk2001sup1.cif

Structure factors: contains datablocks I. DOI: 10.1107/S1600536809027937/fk2001Isup2.hkl

Additional supplementary materials:  crystallographic information; 3D view; checkCIF report

## Figures and Tables

**Table 1:** Hydrogen-bond geometry (Å, °)

*D*—H⋯*A*	*D*—H	H⋯*A*	*D*⋯*A*	*D*—H⋯*A*
N2—H2*A*⋯Cl1^i^	0.86	2.48	3.284 (2)	155

Symmetry code: (i) 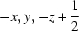.

**Fig. 1.**
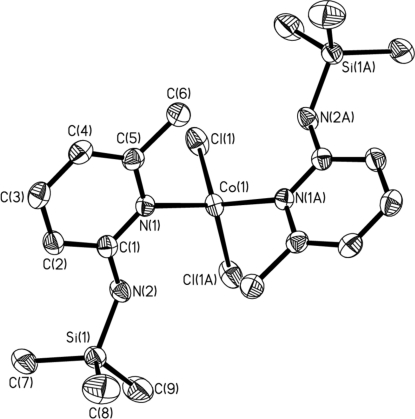
The molecular structure of the title molecule with the atom-numbering scheme. Displacement ellipsoids are drawn at the 40% probability level.

## supplementary crystallographic information

### Comment

The stucture of the title compound, (I), is shown below. The molecule (Co atom) lies on a crystallographic inversion centre. Dimensions are available in the archived CIF. The chemistry of the N-functionalized amido ligands (Liddle and Clegg, 2001; Engelhardt *et al.*, 1988; Kempe, 2000, and references therein) has attracted much interest, and a number of maingroup and transition metal amido complexes with unusual coordination geometry have been isolated. Trimethylsily substituted methyl pyridine ligands have been developed due to their structural features and good catalytic activities (Andrews *et al.*, 2004). Here, we report the synthesis and structure of a new 6-methyl-2-(trimethylsilylamino) pyridine cobalt complex.

The molecular structure is illustrated in Fig. 1. In the complex, the Co atom is four-coordinated in a distorted tetrahedral configuration by two N atoms from two pyridine and two terminal Cl atoms. The bond lengths and angles are within normal ranges. Phenanthridine ring systems are, of course, planar and the dihedral angle between them is A/B = 80.06 (5)°. The compound displays intramolecular N—H···Cl hydrogen bonds (Table 2).

### Experimental

6-Methyl-2-aminopyridine (0.25 g, 2.31 mmol) was added to a solution of LiBu*^n^* (0.81 ml g, 2.31 mmol) in Et_2_O (30 ml) at 0°C. The resulting mixture was then warmed to room temperature and stirred for 3 h. SiMe_3_Cl (0.27 ml, 2.19 mmol) was added at 0°C. The resulting mixture was warmed to room temperature again and stirred for 3 h.CoCl_2_ (0.31 g, 2.39 mmol) was the added at -78°C and the mixture was warmed to room temperature and stirred for 24 h. The volatiles were removed *in vacuo* and the residue was extracted with dichloromethane then filtered. The filtrate was concentrated to give blue crystals (0.79 g, 67%). Anal. Calcd for C_18_H_32_Cl_2_CoN_4_Si_2_(%): C, 44.08; H, 6.58; N 11.42. Found: C, 42.85; H, 6.52; N, 10.99.

### Refinement

H atoms of the methyl groups were derived from Fourier maps (HFIX 137) and allowed to ride during subsequent refinement with C—H = 0.96 Å and *U*_iso_(H) = 1.5*U*_eq_(C). Other hydrogen atoms were refined at calculated positions riding on the C (C–H = 0.95–0.99 Å) or N (N–H = 0.86 Å) atoms with isotropic displacement parameters U_iso_(H) = 1.2U_eq_(C/N).

### Crystal data

**Table:**

[CoCl_2_(C_9_H_16_N_2_Si)_2_]	*F*(000) = 1028
*M**_r_* = 490.49	*D*_x_ = 1.289 Mg m^−^^3^
Monoclinic, *C*2/*c*	Mo *K*α radiation, λ = 0.71073 Å
Hall symbol: -C 2yc	Cell parameters from 2926 reflections
*a* = 14.817 (3) Å	θ = 2.2–260639°
*b* = 12.554 (4) Å	µ = 1.00 mm^−^^1^
*c* = 14.886 (2) Å	*T* = 213 K
β = 114.09 (2)°	Block, blue
*V* = 2527.8 (10) Å^3^	0.30 × 0.30 × 0.20 mm
*Z* = 4	

### Data collection

**Table:**

Bruker SMART APEX CCD area-detector diffractometer	2224 independent reflections
Radiation source: fine-focus sealed tube	1905 reflections with *I* > 2σ(*I*)
graphite	*R*_int_ = 0.020
φ and ω scans	θ_max_ = 25.0°, θ_min_ = 2.2°
Absorption correction: multi-scan (*SADABS*; Sheldrick, 2004)	*h* = −13→17
*T*_min_ = 0.754, *T*_max_ = 0.826	*k* = −14→13
5113 measured reflections	*l* = −17→17

### Refinement

**Table:**

Refinement on *F*^2^	Primary atom site location: structure-invariant direct methods
Least-squares matrix: full	Secondary atom site location: difference Fourier map
*R*[*F*^2^ > 2σ(*F*^2^)] = 0.033	Hydrogen site location: geom and difmap
*wR*(*F*^2^) = 0.089	H-atom parameters constrained
*S* = 1.02	*w* = 1/[σ^2^(*F*_o_^2^) + (0.0559*P*)^2^] where *P* = (*F*_o_^2^ + 2*F*_c_^2^)/3
2224 reflections	(Δ/σ)_max_ = 0.001
127 parameters	Δρ_max_ = 0.47 e Å^−^^3^
0 restraints	Δρ_min_ = −0.19 e Å^−^^3^

### Special details

**Table:**

Geometry. All e.s.d.'s (except the e.s.d. in the dihedral angle between two l.s. planes) are estimated using the full covariance matrix. The cell e.s.d.'s are taken into account individually in the estimation of e.s.d.'s in distances, angles and torsion angles; correlations between e.s.d.'s in cell parameters are only used when they are defined by crystal symmetry. An approximate (isotropic) treatment of cell e.s.d.'s is used for estimating e.s.d.'s involving l.s. planes.
Refinement. Refinement of *F*^2^ against ALL reflections. The weighted *R*-factor *wR* and goodness of fit *S* are based on *F*^2^, conventional *R*-factors *R* are based on *F*, with *F* set to zero for negative *F*^2^. The threshold expression of *F*^2^ > σ(*F*^2^) is used only for calculating *R*-factors(gt) *etc*. and is not relevant to the choice of reflections for refinement. *R*-factors based on *F*^2^ are statistically about twice as large as those based on *F*, and *R*- factors based on ALL data will be even larger.

### Fractional atomic coordinates and isotropic or equivalent isotropic displacement parameters (Å^2^)

**Table:**

	*x*	*y*	*z*	*U*_iso_*/*U*_eq_	
Co1	0.0000	0.78913 (3)	0.2500	0.03732 (16)	
Cl1	0.10398 (5)	0.89853 (5)	0.21598 (5)	0.0592 (2)	
Si1	0.00558 (5)	0.75476 (5)	0.57526 (5)	0.04150 (19)	
N1	0.09602 (12)	0.69403 (12)	0.36153 (13)	0.0331 (4)	
N2	0.04259 (13)	0.76380 (14)	0.47782 (13)	0.0407 (5)	
H2A	0.0166	0.8153	0.4373	0.049*	
C1	0.10695 (15)	0.70085 (15)	0.45668 (16)	0.0352 (5)	
C2	0.18218 (15)	0.64481 (17)	0.53209 (16)	0.0396 (5)	
H2B	0.1894	0.6512	0.5969	0.048*	
C3	0.24442 (16)	0.58107 (17)	0.50937 (17)	0.0437 (6)	
H3A	0.2952	0.5446	0.5588	0.052*	
C4	0.23132 (16)	0.57104 (17)	0.41187 (17)	0.0422 (5)	
H4A	0.2723	0.5262	0.3955	0.051*	
C5	0.15804 (15)	0.62725 (17)	0.34001 (16)	0.0372 (5)	
C6	0.14102 (18)	0.6165 (2)	0.23377 (17)	0.0514 (6)	
H6A	0.1861	0.5649	0.2278	0.077*	
H6B	0.1518	0.6841	0.2096	0.077*	
H6C	0.0743	0.5936	0.1960	0.077*	
C7	0.1062 (2)	0.7875 (2)	0.69626 (19)	0.0632 (7)	
H7A	0.1575	0.7349	0.7127	0.095*	
H7B	0.0804	0.7883	0.7458	0.095*	
H7C	0.1329	0.8564	0.6929	0.095*	
C8	−0.0395 (2)	0.6186 (2)	0.5781 (2)	0.0780 (9)	
H8A	−0.0910	0.6017	0.5152	0.117*	
H8B	−0.0651	0.6141	0.6278	0.117*	
H8C	0.0140	0.5690	0.5930	0.117*	
C9	−0.0937 (2)	0.8548 (3)	0.5436 (2)	0.0819 (10)	
H9A	−0.0685	0.9234	0.5370	0.123*	
H9B	−0.1174	0.8574	0.5947	0.123*	
H9C	−0.1470	0.8356	0.4826	0.123*	

### Atomic displacement parameters (Å^2^)

**Table:**

	*U*^11^	*U*^22^	*U*^33^	*U*^12^	*U*^13^	*U*^23^
Co1	0.0405 (3)	0.0364 (3)	0.0298 (3)	0.000	0.00890 (19)	0.000
Cl1	0.0730 (5)	0.0549 (4)	0.0429 (4)	−0.0265 (3)	0.0167 (3)	−0.0029 (3)
Si1	0.0433 (4)	0.0439 (4)	0.0383 (4)	0.0058 (3)	0.0177 (3)	0.0030 (3)
N1	0.0311 (9)	0.0327 (9)	0.0327 (10)	−0.0001 (7)	0.0102 (8)	−0.0018 (7)
N2	0.0481 (11)	0.0398 (10)	0.0331 (11)	0.0132 (8)	0.0156 (9)	0.0061 (8)
C1	0.0363 (12)	0.0312 (11)	0.0356 (12)	−0.0039 (9)	0.0123 (10)	−0.0017 (9)
C2	0.0403 (12)	0.0419 (12)	0.0334 (12)	0.0002 (10)	0.0117 (10)	0.0041 (10)
C3	0.0363 (12)	0.0412 (12)	0.0473 (15)	0.0044 (10)	0.0107 (11)	0.0063 (11)
C4	0.0353 (12)	0.0425 (12)	0.0484 (14)	0.0032 (10)	0.0168 (11)	−0.0012 (11)
C5	0.0330 (11)	0.0380 (11)	0.0398 (13)	−0.0046 (9)	0.0142 (10)	−0.0061 (10)
C6	0.0444 (13)	0.0660 (16)	0.0431 (14)	0.0052 (12)	0.0170 (11)	−0.0104 (12)
C7	0.0665 (18)	0.0831 (19)	0.0421 (16)	−0.0077 (15)	0.0243 (14)	−0.0082 (14)
C8	0.091 (2)	0.0630 (18)	0.094 (3)	−0.0211 (17)	0.0524 (19)	−0.0041 (17)
C9	0.088 (2)	0.102 (2)	0.071 (2)	0.049 (2)	0.0480 (18)	0.0261 (19)

### Geometric parameters (Å, °)

**Table:**

Co1—N1^i^	2.0681 (17)	C3—H3A	0.9300
Co1—N1	2.0681 (17)	C4—C5	1.369 (3)
Co1—Cl1	2.2701 (7)	C4—H4A	0.9300
Co1—Cl1^i^	2.2701 (7)	C5—C6	1.503 (3)
Si1—N2	1.7512 (19)	C6—H6A	0.9600
Si1—C9	1.843 (3)	C6—H6B	0.9600
Si1—C8	1.843 (3)	C6—H6C	0.9600
Si1—C7	1.856 (3)	C7—H7A	0.9600
N1—C1	1.361 (3)	C7—H7B	0.9600
N1—C5	1.375 (3)	C7—H7C	0.9600
N2—C1	1.370 (3)	C8—H8A	0.9600
N2—H2A	0.8600	C8—H8B	0.9600
C1—C2	1.406 (3)	C8—H8C	0.9600
C2—C3	1.364 (3)	C9—H9A	0.9600
C2—H2B	0.9300	C9—H9B	0.9600
C3—C4	1.389 (3)	C9—H9C	0.9600
			
N1^i^—Co1—N1	109.49 (9)	C3—C4—H4A	120.1
N1^i^—Co1—Cl1	118.51 (5)	C4—C5—N1	121.7 (2)
N1—Co1—Cl1	102.78 (5)	C4—C5—C6	121.01 (19)
N1^i^—Co1—Cl1^i^	102.78 (5)	N1—C5—C6	117.33 (19)
N1—Co1—Cl1^i^	118.51 (5)	C5—C6—H6A	109.5
Cl1—Co1—Cl1^i^	105.54 (4)	C5—C6—H6B	109.5
N2—Si1—C9	103.38 (11)	H6A—C6—H6B	109.5
N2—Si1—C8	108.58 (12)	C5—C6—H6C	109.5
C9—Si1—C8	112.25 (15)	H6A—C6—H6C	109.5
N2—Si1—C7	112.97 (11)	H6B—C6—H6C	109.5
C9—Si1—C7	109.78 (14)	Si1—C7—H7A	109.5
C8—Si1—C7	109.78 (14)	Si1—C7—H7B	109.5
C1—N1—C5	118.28 (17)	H7A—C7—H7B	109.5
C1—N1—Co1	123.35 (13)	Si1—C7—H7C	109.5
C5—N1—Co1	118.11 (14)	H7A—C7—H7C	109.5
C1—N2—Si1	129.45 (15)	H7B—C7—H7C	109.5
C1—N2—H2A	115.3	Si1—C8—H8A	109.5
Si1—N2—H2A	115.3	Si1—C8—H8B	109.5
N1—C1—N2	118.49 (19)	H8A—C8—H8B	109.5
N1—C1—C2	121.22 (19)	Si1—C8—H8C	109.5
N2—C1—C2	120.3 (2)	H8A—C8—H8C	109.5
C3—C2—C1	119.5 (2)	H8B—C8—H8C	109.5
C3—C2—H2B	120.3	Si1—C9—H9A	109.5
C1—C2—H2B	120.3	Si1—C9—H9B	109.5
C2—C3—C4	119.5 (2)	H9A—C9—H9B	109.5
C2—C3—H3A	120.3	Si1—C9—H9C	109.5
C4—C3—H3A	120.3	H9A—C9—H9C	109.5
C5—C4—C3	119.9 (2)	H9B—C9—H9C	109.5
C5—C4—H4A	120.1		
			
N1^i^—Co1—N1—C1	−123.88 (16)	Si1—N2—C1—N1	154.07 (16)
Cl1—Co1—N1—C1	109.30 (15)	Si1—N2—C1—C2	−25.8 (3)
Cl1^i^—Co1—N1—C1	−6.54 (17)	N1—C1—C2—C3	−1.1 (3)
N1^i^—Co1—N1—C5	62.03 (13)	N2—C1—C2—C3	178.84 (19)
Cl1—Co1—N1—C5	−64.80 (14)	C1—C2—C3—C4	−1.2 (3)
Cl1^i^—Co1—N1—C5	179.37 (12)	C2—C3—C4—C5	1.8 (3)
C9—Si1—N2—C1	−172.5 (2)	C3—C4—C5—N1	−0.1 (3)
C8—Si1—N2—C1	−53.1 (2)	C3—C4—C5—C6	−178.9 (2)
C7—Si1—N2—C1	68.9 (2)	C1—N1—C5—C4	−2.0 (3)
C5—N1—C1—N2	−177.26 (17)	Co1—N1—C5—C4	172.35 (15)
Co1—N1—C1—N2	8.7 (2)	C1—N1—C5—C6	176.76 (19)
C5—N1—C1—C2	2.6 (3)	Co1—N1—C5—C6	−8.8 (2)
Co1—N1—C1—C2	−171.44 (14)		

Symmetry codes: (i) −*x*, *y*, −*z*+1/2.

### Hydrogen-bond geometry (Å, °)

**Table:**

*D*—H···*A*	*D*—H	H···*A*	*D*···*A*	*D*—H···*A*
N2—H2A···Cl1^i^	0.86	2.48	3.284 (2)	155

Symmetry codes: (i) −*x*, *y*, −*z*+1/2.
